# Secular Trends in Recurrent Obstetric Anal Sphincter Injuries among 297,190 Women in Norway: A Cohort Study

**DOI:** 10.1007/s00192-025-06417-2

**Published:** 2025-11-14

**Authors:** Katariina Laine, Sari Räisänen

**Affiliations:** 1 Norwegian Research Centre for Women’s Health, Oslo University Hospital, Oslo, Norway; 2 Institute of Clinical Medicine, Faculty of Medicine, University of Oslo, Oslo, Norway; 3 Laurea University of Applied Sciences, Master’s Degree Programs, Vantaa, Finland

**Keywords:** Obstetric anal sphincter injuries, Episiotomy, Epidural, Real-world data, Cohort study

## Abstract

**Introduction and Hypotheses:**

A care bundle to prevent obstetric anal sphincter injuries (OASIS), including manual perineal protection, was launched in Norway in 2005. The study objective was to determine the secular trends of recurrent OASIS in second vaginal birth between 1999 and 2022. OASIS incidence among women without previous OASIS was analyzed for comparison.

**Methods:**

Data for this cohort study were obtained from the Medical Birth Registry of Norway. The study included 297,190 women with singleton pregnancies and two vaginal births during the study period. The outcome was OASIS incidence at second vaginal birth, separately for women with a history of OASIS (recurrent OASIS) and those without. To study the contribution of episiotomy, epidural analgesia, birth mode, birthweight, and maternal age to the secular trends of OASIS, logistic regression analyses with crude and adjusted odds ratios (aORs) with 95% confidence intervals (CIs) were determined.

**Results:**

Of the women with OASIS in their first vaginal birth, 5.4% (609 of 11,205) experienced recurrent OASIS in their second vaginal birth. The incidence of recurrent OASIS decreased from 7.7% in 1999–2002 to 3.5% in 2019–2022, representing a 51% reduction (95% CI 27–67%). Among women without previous OASIS, the OASIS incidence decreased from 1.6% in 1999–2002 to 0.6% in 2019–2022. Obstetric interventions or birthweight did not contribute to these reductions.

**Conclusions:**

The incidence of recurrent OASIS halved in second vaginal births among women with a previous OASIS. This reduction is likely explained by the national care bundle to reduce OASIS, launched in Norway in 2005.

**Supplementary Information:**

The online version contains supplementary material available at 10.1007/s00192-025-06417-2.

## Introduction

Obstetric anal sphincter injuries (OASIS) are severe complications that may occur during vaginal delivery, involving the anal sphincter muscle complex and, in most severe cases, the rectal mucosa [1]. The incidence of OASIS varies significantly across countries, with alarming increases observed in recent decades. A meta-analysis of over 13 million women by Orlando et al. reported an overall incidence of 3.8% for OASIS, with the highest rate (11.8%) in nulliparous women delivered by forceps. OASIS incidence ranged from 2 to 4% in most continents, except in North America, where it was 6.5% [2].

OASIS can lead to significant short- and long-term health problems, including fecal incontinence, chronic perineal pain and dyspareunia, severely impacting daily functioning and overall well-being [3–8]. Recurrence of OASIS in subsequent deliveries is a major concern. A systematic review and meta-analysis of 15 studies involving 700,000 women found that the incidence of recurrent OASIS ranged from 1 to 10% [9]. Risk factors for OASIS, such as operative vaginal birth, prolonged second stage of labor, and high birthweight, are consistent in both primary and recurrent cases [9–11].

Although risk factors for recurrent OASIS are well-documented, more research is needed to develop effective prevention strategies. Some studies suggest that episiotomy, particularly lateral and mediolateral, may reduce the risk of OASIS [12], although findings are conflicting. A multicenter study from D’Souza et al. found an 80% reduction in recurrent OASIS risk with mediolateral episiotomy use [13], while Barba et al. reported that median or mediolateral episiotomy was not protective against recurrent OASIS [9].

In Norway, a national care bundle was launched in 2005–2006, emphasizing manual perineal protection, selective use of mediolateral or lateral episiotomy, and careful communication with the delivering woman during labor. This significantly reduced OASIS incidence in both spontaneous and operative vaginal deliveries [11, 14–18]. Similar results have been reported in Denmark, the Netherlands, the UK, and Palestine [19–22]. However, to the best of our knowledge there is no previous evidence linking manual perineal support to the prevention of recurrent OASIS.

The main aim of this study was to analyze secular trends in the incidence of recurrent OASIS in second vaginal births from 1999 to 2022 in Norway. OASIS incidence in second pregnancy among women without previous OASIS was analyzed for comparison.

## Materials and Methods

This population-based observational study utilized prospectively collected real-world data gathered from the Medical Birth Registry of Norway (MBRN) between 1999 and 2022. The study design was evaluated and approved by the Regional Committee for Medical Research Ethics of Southeast Norway (2015/681, renewed in 2017, 2019, 2020, and 2023). All parts of the study followed Norwegian Health Research legislation.

In Norway, all maternity hospitals are publicly funded, and no private birth care hospitals are available. Only 0.2% of the annual births are planned home births. Antenatal care is standardized and offered free of charge for all residents. Midwives independently manage pregnancies, labors, and births that are considered normal. Physicians become involved only if the attending midwife determines that their expertise is needed. In midwife-led births where an obstetrician is not required, midwives independently evaluate the need for episiotomy during the second stage of labor. The need for epidural analgesia is evaluated by the attending midwife together with the woman giving birth and the anesthesiologist on duty.

For the present study, we analyzed MBRN data collected over a 24-year period from 1999 to 2022. The MBRN is a mandatory quality registry that records all births (including home births). The MBRN, as the register keeper, pseudonymized the data, and any contact between the researchers and the study participants was not possible or necessary.

Information on the maternal pre-pregnancy health status, reproductive history, follow-up visits, and complications during pregnancy is prospectively recorded on a standardized pregnancy health card during antenatal healthcare appointments, similarly throughout the country. Interventions during labor and birth, and the health and condition of the newborn are recorded during and after birth by the attending midwife and doctor, and a mandatory notification is sent to the MBRN. This information is digitally transferred to the MBRN immediately after birth. The health statuses of the woman and newborn are also recorded and registered at the MBRN during the hospital stay, including observations made by a pediatrician or a perinatologist. The MBRN validates the data against other health registries, the population registry, and the death registry.

Women who had their first and second vaginal births during the study period from 1999 to 2022, with singleton fetuses (*N* = 297,190), were included in the study population. Cesarean sections (CS), multiple pregnancies, and pregnancies before gestational week 22 + 0 were excluded. The study population was divided into two groups: Study group 1 consisted of women with OASIS in their first vaginal birth (*n* = 11,205), and study group 2 consisted of women who did not have OASIS in their first vaginal birth (*n* = 285,985) (Table 1). A flow chart designed to illustrate the formation of the study population is reported in Table 1.

**Table 1:** Flow chart for the study population

**Study population in 1999–2022**	
Women with their first and second birth during the study period *N* = 373,024	
Women with first birth by cesarean excluded *n* = 56,344	
**First birth vaginal, *n* = 316,680**	
First birth with OASIS, *n* = 14,430	First birth without OASIS *n* = 302,250
**Second birth**	**Second birth:**
Second birth planned cesarean excluded 17.0% (2458 of 14,430)	Second birth planned cesarean excluded 2.2% (6521 of 302,250)
Second birth planned vaginal 83% (11,972 of 14,430)	Second birth planned vaginal 97.8% (295,729 of 302,250)
Second birth intrapartum cesarean excluded 6.4% (767 of 11,972)	Second birth intrapartum cesarean excluded 3.3% (9.744 of 295,729)
**First and second birth vaginal**, ***n* = 297,190**	
**Study group 1** OASIS in the first birth, *n* = 11,205	**Study group 2** No OASIS in the first birth, *n* = 285,985
OASIS in the second birth, *n* = 609	OASIS in the second birth, *n* = 2644

The outcome variable was OASIS in second vaginal birth, defined as an injury in either the external anal sphincter muscle or both the internal and external muscles. Injuries classified as degrees 3 A, 3B, 3 C, and 4 were merged into a single variable (OASIS) in the MBRN. OASIS is registered dichotomously (yes or no) in the MBRN, and we do not have more detailed information available about the degree of OASIS (third or fourth). To study the secular trends of recurrent OASIS, the study period (1999–2022) was divided into six 4-year time periods: 1999–2002, 2003–2006, 2007–2010, 2011–2014, 2015–2018, and 2019–2022. The first time period was used as the reference in the analyses.

OASIS risk factors included in the statistical analyses as confounders were identified on the basis of previous literature [2, 9, 11, 23]. Episiotomy and epidural analgesia were dichotomized to either yes or no. In line with international agreement, episiotomy use is recommended by indication only, and either the lateral or mediolateral type of episiotomy is used in Norway [14, 24]. Birthweight in grams was categorized into four groups: < 3000, 3000–3499 (reference), 3500–3999, and ≥ 4000. Maternal age in years was categorized into five groups: < 25, 25–29 (reference), 30–34, 35–39, and ≥ 40.

### The Intervention

The Norwegian Directorate of Health launched a comprehensive national care bundle in 2005. The care bundle was gradually adopted by maternity units throughout Norway over time. This care bundle emphasized manual protection of the perineum with two hands: one hand on the baby’s head controlling the speed of head expulsion and the other hand supporting perineum, (illustrated in Photo 1, supplementary material), judicious episiotomy use with either a lateral or mediolateral technique, and enhanced communication with the woman to avoid pushing during the last phase of the second stage when the fetal head is crowning. The care bundle also included providing education on the diagnosis and primary repair of OASIS.

### Statistical Analyses

The incidence of recurrent OASIS was calculated on the basis of six 4-year time periods from 1999 to 2022, considering maternal and fetal demographic and obstetric factors. For comparison, similar calculations were conducted for women without previous OASIS in their first vaginal birth.

Logistic regression analyses, using a backward elimination method to select a subset of explanatory variables for the model, were conducted to determine the OASIS incidence, considering previously reported risk factors and time periods. Both the crude odds ratios (ORs) and adjusted odds ratios (aORs) with 95% confidence intervals (CI) were determined for all variables.

To examine the contribution of episiotomy, epidural, birth mode, and birthweight, as independent variable on secular trends in recurrent OASIS, we created four different models, with each of the previously mentioned covariates added individually to the crude model. Similar analyses were conducted for the study group 2 (no previous OASIS). SPSS software (version 29, IBM) was used for the analyses.

### Missing Data

The newborn birthweight was missing in 0.02% (*n* = 70) births, and these were excluded from the logistic regression analyses.

## Results

The study population consisted of 297,190 women who had their first and second vaginal births during the study period from 1999 to 2022. There were 11,205 women with an OASIS in their first vaginal birth (study group 1) and 285,985 women without previous OASIS in the first vaginal (study group 2) (Table 2). Table 2 reports the use of obstetric interventions, birthweight, and maternal age during the study period, separately for study groups 1 and 2.

**Table 2:** Obstetric interventions, birth mode, birthweight, and maternal age in women with second vaginal birth, with or without a previous OASIS in their first vaginal birth in Norway from 1999 to 2022, *N* = 297,190

Characteristic	Study group 1 Previous OASIS *n* = 11,205	Study group 2 No previous OASIS *n* = 285,985
	% (*n*)	% (*n*)
Time periods		
1999–2002	7.6 (856)	5.0 (14,163)
2003–2006	26.4 (2963)	16.1 (45,909)
2007–2010	24.5 (2747)	19.0 (54,237)
2011–2014	17.2 (1930)	20.1 (57,549)
2015–2018	13.6 (1529)	20.2 (57,766)
2019–2022	10.5 (1180)	19.7 (56,361)
Episiotomy		
No	75.1(8412)	93.3 (266,960)
Yes	24.9 (2793)	6.7 (19,025)
Epidural analgesia		
No	80.5 (9021)	79.0 (225,995)
Yes	19.5 (2184)	21.0 (59,990)
Birth mode		
Spontaneous vaginal	95.9 (10,748)	97.1 (277,629)
Vacuum extraction	3.5 (388)	2.5 (7159)
Forceps	0.6 (69)	0.4 (1197)
Birthweight, grams		
< 3000	7.2 (808)	8.7 (24,895)
3000–3499	25.8 (2894)	29.3 (83,697)
3500–3999	39.7 (4448)	39.5 (112,984)
4000–4499	22.1 (2479)	18.6 (53,079)
≥ 4500	5.1 (573)	3.9 (11,263)
Missing value	0.03 (3)	0.02 (67)
Maternal age, years		
< 25	7.0 (748)	10.4 (29,728)
25–29	32.5 (3643)	34.0 (97,120)
30–34	43.5 (4880)	39.6 (113,277)
35–39	15.2 (1794)	14.2 (40,501)
≥ 40	1.7 (194)	1.9 (5359)

Study group 1: Women with second vaginal births with OASIS in their first vaginal birth

Study group 2: Women with second vaginal births without OASIS in their first vaginal birth

*OASIS *obstetric anal sphincter injuries

In study group 1 (women with previous OASIS), the overall incidence of recurrent OASIS was 5.4% (609 of 11,205) (Table 3). In study group 2 (women without previous OASIS), the overall incidence of OASIS was 0.9% (2644 of 285,985) (Table 3). OASIS incidences by maternal and fetal characteristics, obstetric interventions, and birth mode are reported in Table 3.

**Table 3:** OASIS incidence in second vaginal birth, among women with or without previous OASIS in their first vaginal births, in Norway from 1999 to 2022

Characteristic	Study group 1 Previous OASIS *n* = 11,205	Study group 2 No previous OASIS *n* = 285,985
	% (*n*)	% (*n*)
OASIS	5.4 (609 of 11,205)	0.9 (2644 of 285,985)
Time periods		
1999–2002	7.7 (66 of856)	1.6 (232 of 14,163)
2003–2006	8.2 (243 of 2963)	1.6 (720 of 45,909)
2007–2010	4.4 (120 of 2747)	0.9 (513 of 54,237)
2011–2014	4.8 (92 of 1930)	0.8 (458 of 57,549)
2015–2018	3.1 (47 of 1529)	0.6 (365 of 57,766)
2019–2022	3.5 (41 of 1180)	0.6 (356 of 56,361)
Episiotomy		
No	5.7 (478 of 8412)	0.9 (2,342 of 266,960)
Yes	4.7 (131 of 2793)	1.6 (302 of 19,025)
Epidural analgesia		
No	5.7 (512 of 9021)	0.9 (2102 of 225,995)
Yes	4.4 (97of 2184)	0.9 (542 of 59,990)
Birth mode		
Spontaneous vaginal	5.3 (569 of 10,748)	0.9 (2370 of 277,629)
Vacuum extraction	8.0 (31 of 388)	3.1 (224 of 7159)
Forceps	13.0 (9 of 69)	4.5 (54 of 1197)
Birthweight, grams		
< 3000	2.2 (18 of 808)	0.3 (63 of 24,895)
3000–3499	3.4 (99 of 2894)	0.5 (416 of 83,697)
3500–3999	4.9 (219 of 4448)	0.9 (984 of 112,984)
4000–4499	8.2 (203 of 2479)	1.6 (833 of 53,079)
≥ 4500	12.2 (70 of 573)	3.1 (348 of 11,263)
Missing value	0.0 (0 of 3)	0.0 (0 of 67)
Maternal age, years		
< 25	4.7 (37 of 748)	0.6 (171 of 29,728)
25–29	5.5 (201 of 3673)	0.8 (807 of 97,120)
30–34	5.6 (271 of 4880)	1.1 (1,193 of 113,277)
35–39	5.0 (86 of 1794)	1.0 (424 of 40,501)
≥ 40	7.2 (14 of 194)	0.9 (49 of 5359)

Study group 1: Women with second vaginal births with OASIS in their first vaginal birth

Study group 2: Women with second vaginal births without OASIS in their first vaginal birth

*OASIS* obstetric anal sphincter injuries

**Fig. 1:**
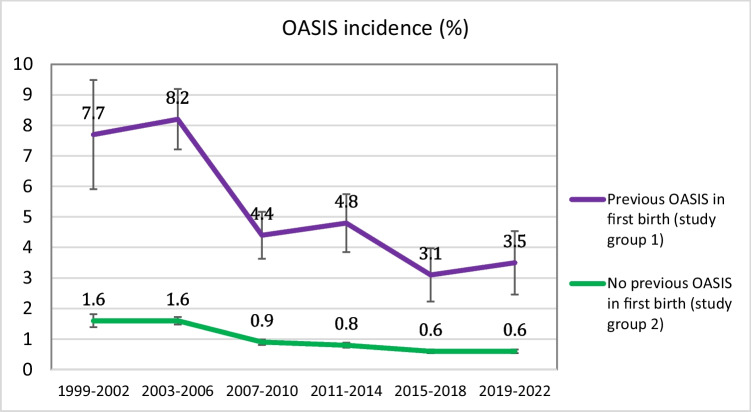
OASIS incidence (%) in study group 1 (previous OASIS) and study group 2 (no previous OASIS) in the time periods, with confidence intervals. Study group 1: Women with second vaginal births with OASIS in their first vaginal birth. Study group 2: Women with second vaginal births without OASIS in their first vaginal birth. *OASIS* obstetric anal sphincter injuries

In study group 1, the incidence of recurrent OASIS decreased gradually over the entire study period, from 7.7% in 1999–2002 to 3.5% in 2019–2022 (Table 3, Fig. 1), representing a 51% decrease (aOR 0.49, 95% CI 0.33–0.73) (Table 4). In study group 2, the incidence of OASIS decreased from 1.6% to 0.6% (Table 3, Fig. 1), representing a 63% decrease (aOR 0.37, 95% CI 0.31–0.45) (Table 4).

**Table 4:** Crude odds ratios (OR) and adjusted ORs for OASIS in second vaginal births, among women with or without a previous OASIS in their first vaginal birth in Norway in 1999–2022, *N* = 297,190

Characteristic	Study group 1 Previous OASIS *n* = 11,205		Study group 2 No previous OASIS *n* = 285,985	
	ORs (95% CI)	aORs (95% CI)	ORs (95% CI)	aORs (95% CI)
Time periods				
1999–2002	Ref	Ref	Ref	Ref
2003–2006	1.07 (0.81–1.42)	1.08 (0.81–1.44)	0.96 (0.82–1.11)	0.93 (0.80–1.09)
2007–2010	0.55 (0.40–0.75)	0.60 (0.44–0.81)	0.57 (0.49–0.67)	0.58 (0.49–0.67)
2011–2014	0.60 (0.43–0.83)	0.64 (0.46–0.89)	0.48 (0.41–0.57)	0.48 (0.41–0.57)
2015–2018	0.38 (0.26–0.56)	0.43 (0.29–0.63)	0.38 (0.32–0.45)	0.38 (0.32–0.45)
2019–2022	0.43 (0.29–0.64)	0.49 (0.33–0.73)	0.38 (0.32–0.45)	0.37 (0.31–0.45)
Episiotomy				
No	Ref	Ref	Ref	
Yes	0.82 (0.67–0.99)	0.79 (0.64–0.96)	1.82 (1.62–2.06)	NS
Epidural analgesia				
No	Ref	Ref	Ref	Ref
Yes	0.77 (0.62–0.96)	0.76 (0.60–0.95)	0.97 (0.88–1.07)	0.88 (0.79–0.97)
Birth mode				
Spontaneous vaginal	Ref	Ref	Ref	Ref
Vacuum extraction	1.55 (1.07–2.24)	1.57 (1.06–2.31)	3.68 (3.20–4.24)	3.26 (2.83–3.79)
Forceps	2.68 (1.33–5.43)	3.62 (1.75–7.50)	5.49 (4.16–7.23)	5.88 (4.43–7.79)
Birthweight, grams				
< 3000	0.64 (0.39–1.07)	0.64 (0.39–1.07)	0.51 (0.39–0.66)	0.51 (0.39–0.67)
3000–3499	Ref	Ref	Ref	Ref
3500–3999	1.46 (1.15–1.86)	1.45 (1.13–1.85)	1.76 (1.57–1.97)	1.71 (1.53–1.92)
4000–4499	2.52 (1.97–3.22)	2.44 (1.91–3.13)	3.19 (2.84–3.59)	3.02 (2.68–3.40)
≥ 4500	3.93 (2.85–5.41)	3.69 (2.66–5.10)	6.38 (5.53–7.37)	5.75 (4.97–6.65)
Maternal age, years				
< 25	0.85 (0.59–1.22)	NS	0.69 (0.59–0.82)	0.69 (0.58–0.81)
25–29	Ref		Ref	Ref
30–34	1.01 (0.84–1.22)	NS	1.27 (1.16–1.39)	1.28 (1.17–1.41)
35–39	0.91 (0.70–1.18)	NS	1.26 (1.12–1.42)	1.33 (1.18–1.50)
≥ 40	1.33 (0.76–2.33)	NS	1.10 (0.82–1.47)	1.26 (0.94–1.69)

Logistic regression analyses with the backward elimination method were used

Study group 1: Women with second vaginal births with OASIS in their first vaginal birth

Study group 2: Women with second vaginal births without OASIS in their first vaginal birth

*OASIS* obstetric anal sphincter injuries

*Ref* reference category

*NS* nonsignificant

In study group 1, using either a mediolateral or lateral episiotomy (24.9%, 2793 of 11,205) (Table 3) was associated with a 21% decrease in the incidence of OASIS (aOR 0.79, 95% CI 0.64–0.96) compared to births with no episiotomy (Table 4).

In study group 2, the use of either a mediolateral or lateral episiotomy (6.7%, 19,025 of 285,985) (Table 3) was not significantly associated with the incidence of OASIS compared to births with no episiotomy after adjustment for confounders (Table 4).

In study group 1, a 1.6-fold OASIS incidence was associated with vacuum extraction (aOR 1.57, 95% CI 1.06–2.31) and a 3.6-fold OASIS incidence was associated with forceps (aOR 3.62, 95% CI 1.75–7.50), compared to spontaneous vaginal birth.

In study group 2, a 3.3-fold OASIS incidence was associated with vacuum extraction (aOR 3.26, 95% CI 2.83–3.79), and a 5.9-fold OASIS incidence was associated with forceps delivery (aOR 5.88, 95% CI 4.43–7.79), compared to spontaneous vaginal birth (Table 4).

In both study groups, higher birthweight, specifically over 4000 g and over 4500 g, was associated with a two- to sixfold increased OASIS incidence compared with a birthweight of 3000–3499 g (Table 4). Women with epidural analgesia had a 24% (aOR 0.76, 95% CI 0.60–0.95) lower OASIS incidence in study group 1 and a 12% (aOR 0.88, 95% CI 0.79–0.97) lower incidence of OASIS in study group 2 compared to women without epidural analgesia (Table 4). Table 5 shows that obstetric interventions (models 1, 2, and 3) and birthweight (model 4) had either no or negligible effect on the ORs of OASIS in study group 1 and 2 (ORs of OASIS in models 1–4 compared to crude model, Table 5). The odds for OASIS remained the same or the changes were insignificant after including episiotomy (Table 5, model 1), epidural analgesia (Table 5, model 2), birth mode (Table 5, model 3), and birthweight (Table 5, model 4) one by one in the crude model (Table 5).

**Table 5:** Results of logistic regression analyses to study the contribution of obstetric interventions and fetal birthweight to the incidence of obstetric anal sphincter injuries (OASIS) in second vaginal birth, with or without a previous OASIS in their first vaginal birth

Characteristic	Crude analysis OR (95% CI)	Model 1 aOR (95% CI)	Model 2 aOR (95% CI)	Model 3 aOR (95% CI)	Model 4 aOR (95% CI)
Study group 1 Previous OASIS *n* = 11,205					
Time periods					
1999–2002	Ref	Ref	Ref	Ref	Ref
2003–2006	1.07 (0.81–1.42)	1.07 (0.80–1.41)	1.07 (0.81–1.42)	1.06 (0.80–1.40)	1.10 (0.83–1.47)
2007–2010	0.55 (0.40–0.75)	0.55 (0.40–0.75)	0.55 (0.40–0.75)	0.54 (0.40–0.73)	0.60 (0.44–0.82)
2011–2014	0.60 (0.43–0.83)	0.60 (0.43–0.83)	0.60 (0.44–0.84)	0.58 (0.42–0.81)	0.65 (0.47–0.90)
2015–2018	0.38 (0.26–0.56)	0.38 (0.26–0.56)	0.39 (0.30–0.66)	0.38 (0.26–0.55)	0.42 (0.29–0.62)
2019–2022	0.43 (0.29–0.64)	0.44 (0.29–0.65)	0.44 (0.30–0.66)	0.43 (0.29–0.63)	0.47 (0.32–0.71)
Study group 2 No previous OASIS *n* = 285,985					
Time periods					
1999–2002	Ref	Ref	Ref	Ref	Ref
2003–2006	0.96 (0.82–1.11)	0.97 (0.83–1.12)	0.96 (0.82–1.11)	0.95 (0.82–1.11)	0.97 (0.84–1.13)
2007–2010	0.57 (0.49–0.67)	0.58 (0.50–0.68)	0.57 (0.49–0.67)	0.56 (0.48–0.66)	0.61 (0.52–0.71)
2011–2014	0.48 (0.41–0.57)	0.49 (0.42–0.58)	0.48 (0.41–0.56)	0.47 (0.40–0.55)	0.52 (0.44–0.61)
2015–2018	0.38 (0.32–0.45)	0.39 (0.33–0.46)	0.38 (0.32–0.45)	0.37 (0.32–0.44)	0.41 (0.35–0.48)
2019–2022	0.38 (0.32–0.45)	0.39 (0.33–0.46)	0.38 (0.32–0.45)	0.37 (0.32–0.44)	0.40 (0.34–0.47)

Study group 1: Women with second vaginal births with OASIS in their first vaginal birth

Study group 2: Women with second vaginal births without OASIS in their first vaginal birth

*aOR* adjusted odds ratio, *Ref* reference category

Model 1. Odds ratios of OASIS adjusted by episiotomy (crude analysis + episiotomy)

Model 2. Odds ratios of OASIS adjusted by epidural analgesia (crude analysis + epidural analgesia)

Model 3. Odds ratios of OASIS adjusted by birth mode (crude analysis + birth mode)

Model 4. Odds ratios of OASIS adjusted by fetal birthweight (crude analysis + fetal birthweight)

Table 5 shows that obstetric interventions (episiotomy in model 1, epidural analgesia in model 2, and birth mode in model 3) and birthweight (in model 4) had either no or only a negligible effect on the odds ratios (ORs) of OASIS (the ORs remained largely unchanged or showed only minimal variation) when compared to the crude model in both study groups 1 and 2. This indicates that episiotomy, epidural analgesia, birth mode, or birthweight did not influence the declining OASIS incidence observed across the five later time periods compared to the first.

The OASIS incidence was 5.9-fold (OR 5.88, 95% CI 5.37–6.43) in women with OASIS in their previous vaginal birth compared to those women with no OASIS in their previous vaginal birth (data not shown).

Of the women with previous OASIS, 17.0% were delivered with a planned CS, while 2.2% of women without previous OASIS were delivered by planned cesarean (Table 1).

## Discussion

### Main Findings

The main and novel finding of this study was that the incidence of recurrent OASIS in second vaginal births, following a first vaginal birth complicated by OASIS, decreased from 7.7% in 1999–2002 to 3.5% in 2019–2022. This represents a 51% reduction (95% CI 27% to 67%). During the same period, a 63% reduction in OASIS incidence (95% CI 55% to 69%) was observed in second vaginal births among women who did not experience OASIS during their first vaginal birth, with rates declining from 1.6% to 0.6%. The declining trend in OASIS incidence continued for many years after the implementation of the care bundle, demonstrating the long-term sustainability of the national strategy.

This reduction is likely explained by the implementation of manual perineal protection as part of a national care bundle launched in 2005 to prevent OASIS. Obstetric interventions such as episiotomy and epidural analgesia, as well as birth mode and birthweight, had either no effect or only a negligible impact on the association between time periods and OASIS incidence. To the best of our knowledge, an association between manual perineal protection and recurrent OASIS has not been reported in previous studies.

### Strength and Limitations

The primary strength of this study was the use of prospectively collected, population-based, real-world data and a long-term follow-up over a 24-year period. Another strength was that we compared OASIS incidence in a second vaginal birth separately for those with and without a previous OASIS to obtain information on the recurrence of OASIS in second vaginal births [25].

The main limitation is the potential for inaccuracies and errors in register-based data. However, data on OASIS in the MBRN have been previously validated [26], and the amount of missing information was notably low. Another limitation is that the use of manual perineal protection is not recorded in the MBRN; however, we can report that a nationwide care bundle, which included manual perineal protection, was launched in 2005 and gradually adopted in the entire country during the study period. We cannot find any other explanation for the reduced OASIS incidences in Norway, as previously reported in several studies from Norway [14–18, 27, 28]. Lacking information of the type of episiotomy is also a limitation in the study. However, midline episiotomy has never been recommended in Norway, only mediolateral or lateral techniques are recommended, when episiotomy is indicated.

### Interpretation

The odds of OASIS in each time period remained unchanged or showed only minor changes when adjusted individually for obstetric interventions, mode of birth, or birthweight in the crude model (Table 5). On this basis, we suggest that the implementation of manual perineal protection was the key factor in reducing the incidence of recurrent OASIS in second vaginal births, as well as the OASIS incidence among women without a previous OASIS. The present results are in line with previous studies on the decreasing trend of OASIS in spontaneous vaginal births in nulliparous women [18] and operative vaginal births during the same study period in Norway [14]. Previous studies from Denmark, the Netherlands, the UK, and Palestine have reported a decline in OASIS incidence associated with the implementation of manual perineal protection as a clinical routine [19–22]. A recent study from the UK reported that use of manual perineal support did not interfere with birth experience [29].

A history of OASIS in the first vaginal birth appears to be a major risk factor for OASIS in subsequent vaginal birth. The incidence of recurrent OASIS was 5.9-fold (OR 5.88, 95% CI 5.37–6.43) compared to OASIS occurring in second vaginal births without a history of OASIS. To the best of our knowledge, this association has been less studied, with most previous studies focusing only on the incidence and risk factors for recurrent OASIS [9].

The systematic review and meta-analysis by Barba et al., from 2021, reported an OASIS incidence ranging from 1 to 10% and noted that most previous studies focused on risk factors rather than preventive interventions [9]. Barba et al. found conflicting results regarding the association between mediolateral or median episiotomy and recurrent OASIS. Our results showed a 21% (95% CI 4–36%) decrease in recurrent OASIS incidence in women with mediolateral or lateral episiotomy compared to no episiotomy in their second vaginal birth. This suggests that mediolateral or lateral episiotomy was protective against recurrent OASIS. Among women experiencing a second vaginal birth without a history of OASIS, the association between OASIS and episiotomy was not statistically significant after adjusting for obstetric interventions and birthweight in the logistic regression analysis (Table 4). This association is likely explained by confounding by indication, suggesting that episiotomy was performed when perineal tearing appeared imminent. However, in both study groups, those with previous OASIS and those without, the incidence of OASIS decreased by 50 to 60% during the study period, regardless of episiotomy use. In both groups, the decline began between 2007 and 2010, shortly after the implementation of manual perineal protection in 2005 (Table 4).

The generalizability of our findings is strong, as manual perineal protection is a low-cost intervention that can be implemented in any setting where vaginal births are managed. It does not require expensive equipment or medication. Importantly, our results suggest that this intervention is effective not only in reducing the overall incidence of OASIS but also in preventing recurrent OASIS. However, to achieve positive results implementing manual perineal protection intervention requires not only thorough theoretical and hands-on practical training, as well as the correct technique [14, 19, 30], but also staff compliance to ensure low levels of OASIS [21].

### Conclusion

Previous studies have shown that implementing manual perineal protection reduced OASIS incidence in general [11, 17, 19, 20], in nulliparous women with spontaneous vaginal births [18], and in instrumental vaginal births [14]. The novel finding of the present study was that after launching a national care bundle to reduce the high and increasing OASIS incidence by implementing manual perineal protection [15–17, 27], the incidence of recurrent OASIS was also significantly reduced, from 7.7% in 1999–2002 to 3.5% in 2019–2022.

Our results suggest that providing comprehensive education on manual perineal protection methods can effectively reduce the OASIS likely due to extensive staff training. It is essential for future research to include a detailed description of the intervention being tested and the methods used to train the labor room staff.

## Supplementary Information

Below is the link to the electronic supplementary material.

Supplementary Fig. 1 The photo illustrates the methods of manual perineal protection with two hands. (JPEG 13 KB)

## Data Availability

The dataset used in this study was obtained from the Norwegian Medical Birth Registry. Due to the sensitive nature of the data, which includes personal health information, it is not publicly available. Data access and processing must comply with Norwegian Health Research legislation. Researchers interested in using the data for secondary analysis may contact the corresponding author and seek approval from the relevant Norwegian authority responsible for granting access to registry-based health data.

## References

[1] Sultan AH. Editorial: Obstetrical perineal injury and anal incontinence. Clin Risk. 1999. 10.1177/135626229900500601.

[2] Orlando A, Thomas G, Murphy J, Hotouras A, Bassett P, Vaizey C. A systematic review and a meta-analysis on the incidence of obstetric anal sphincter injuries during vaginal delivery. Colorectal Dis. 2024;26:227–42. 10.1111/codi.16831. 38131640 10.1111/codi.16831

[3] Nilsson IEK, Åkervall S, Molin M, Milsom I, Gyhagen M. Symptoms of fecal incontinence two decades after no, one, or two obstetrical anal sphincter injuries. Am J Obstet Gynecol. 2021;224:276.e1-276.e23. 10.1016/j.ajog.2020.08.051. 32835724 10.1016/j.ajog.2020.08.051

[4] Baumann S, Staudt A, Horesh D, Eberhard-Gran M, Garthus-Niegel S, Horsch A. Perineal tear and childbirth-related posttraumatic stress: a prospective cohort study. Acta Psychiatr Scand. 2023. 10.1111/acps.13595. 37550260 10.1111/acps.13595

[5] Lindqvist M, Lindberg I, Nilsson M, Uustal E, Persson M. Struggling to settle with a damaged body” – a Swedish qualitative study of women’s experiences one year after obstetric anal sphincter muscle injury (OASIS) at childbirth. Sex Reprod Healthc. 2019;19:36–41. 10.1016/j.srhc.2018.11.002. 30928133 10.1016/j.srhc.2018.11.002

[6] Fodstad K, Staff AC, Laine K. Sexual activity and dyspareunia the first year postpartum in relation to degree of perineal trauma. Int Urogynecol J. 2016;27:1513–23. 10.1007/s00192-016-3015-7. 27185318 10.1007/s00192-016-3015-7

[7] Gommesen D, Nøhr E, Qvist N, Rasch V. Obstetric perineal tears, sexual function and dyspareunia among primiparous women 12 months postpartum: a prospective cohort study. BMJ Open. 2019;9:e032368. 10.1136/bmjopen-2019-032368. 31848167 10.1136/bmjopen-2019-032368 PMC6937116

[8] Laine K, Skjeldestad FE, Sandvik L, Staff AC. Prevalence and risk indicators for anal incontinence among pregnant women. ISRN Obstet Gynecol. 2013;2013:947572. 10.1155/2013/947572. 23819058 10.1155/2013/947572 PMC3681258

[9] Barba M, Bernasconi DP, Manodoro S, Frigerio M. Risk factors for obstetric anal sphincter injury recurrence: a systematic review and meta-analysis. Int J Gynaecol Obstet. 2022;158:27–34. 10.1002/ijgo.13950. 34559892 10.1002/ijgo.13950 PMC9298380

[10] Baghestan E, Irgens LM, Bordahl PE, Rasmussen S. Risk of recurrence and subsequent delivery after obstetric anal sphincter injuries. BJOG. 2012;119:62–9. 10.1111/j.1471-0528.2011.03150.x 21985470 10.1111/j.1471-0528.2011.03150.x

[11] Laine K, Skjeldestad FE, Sandvik L, Staff AC. Incidence of obstetric anal sphincter injuries after training to protect the perineum: cohort study. BMJ Open. 2012;2:e001649. 10.1136/bmjopen-2012-001649. 23075573 10.1136/bmjopen-2012-001649 PMC3488722

[12] van Bavel J, Ravelli A, Abu-Hanna A, Mol B, Roovers J, de Leeuw J. The effect of a mediolateral episiotomy on the recurrence of obstetrical anal spincter injury(OASI): an analysis of a national registry. Int Urogynecol J 2018;29. 10.1111/1471-0528.16263 32285571

[13] D’Souza JC, Monga A, Tincello DG, Sultan AH, Thakar R, Hillard TC, et al. Maternal outcomes in subsequent delivery after previous obstetric anal sphincter injury (OASI): a multi-centre retrospective cohort study. Int Urogynecol J. 2020;31:627–33. 10.1007/s00192-019-03983-0. 31230097 10.1007/s00192-019-03983-0 PMC7093337

[14] Fodstad K, Laine K, Räisänen S. Obstetric anal sphincter injuries during instrumental vaginal delivery: an observational study based on 18-years of real-world data. BJOG. 2024(13). 10.1111/1471-0528.17914. 10.1111/1471-0528.17914 39030798

[15] Hals E, Øian P, Pirhonen T, Gissler M, Hjelle S, Nilsen EB, et al. A multicenter interventional program to reduce the incidence of anal sphincter tears. Obstet Gynecol. 2010;116:901–8. 10.1097/AOG.0b013e3181eda77a. 20859154 10.1097/AOG.0b013e3181eda77a

[16] Eggebø TM, Rygh AB, von Brandis P, Skjeldestad FE. Prevention of obstetric anal sphincter injuries with perineal support and lateral episiotomy: a historical cohort study. Acta Obstet Gynecol Scand. 2023. 10.1111/aogs.14742. 38053429 10.1111/aogs.14742 PMC10867358

[17] Laine K, Rotvold W, Staff AC. Are obstetric anal sphincter ruptures preventable?- Large and consistent rupture rate variations between the Nordic countries and between delivery units in Norway. Acta Obstet Gynecol Scand. 2013;92:94–100. 10.1111/aogs.12024. 23034015 10.1111/aogs.12024

[18] Laine K, Fodstad K, Räisänen S. Obstetric anal sphincter injuries in spontaneous vaginal births in nulliparous pregnant individuals: a 21-year cohort study based on real-world data. Am J Obstet Gynecol. 2025. 10.1016/j.ajog.2025.06.014. 40513930 10.1016/j.ajog.2025.06.014

[19] Rasmussen OB, Yding A, Andersen CS, Boris J, Lauszus FF. Which elements were significant in reducing obstetric anal sphincter injury? A prospective follow-up study. BMC Pregnancy Childbirth. 2021;21:781. 10.1186/s12884-021-04260-z. 34794417 10.1186/s12884-021-04260-z PMC8600779

[20] Gurol-Urganci I, Bidwell P, Sevdalis N, Silverton L, Novis V, Freeman R, et al. Impact of a quality improvement project to reduce the rate of obstetric anal sphincter injury: a multicentre study with a stepped-wedge design. BJOG. 2021;128:584–92. 10.1111/1471-0528.16396. 33426798 10.1111/1471-0528.16396 PMC7818460

[21] De Meutter L, van Heesewijk AD, van der Woerdt-Eltink I, de Leeuw JW. Implementation of a perineal support programme for reduction of the incidence of obstetric anal sphincter injuries and the effect of non-compliance. Eur J Obstet Gynecol Reprod Biol. 2018;230:119–23. 10.1016/j.ejogrb.2018.09.021. 30253277 10.1016/j.ejogrb.2018.09.021

[22] Ali-Masri H, Hassan S, Fosse E, Zimmo KM, Zimmo M, Ismail KMK, et al. Impact of electronic and blended learning programs for manual perineal support on incidence of obstetric anal sphincter injuries: a prospective interventional study. BMC Med Educ. 2018;18:258. 10.1186/s12909-018-1363-3. 30419884 10.1186/s12909-018-1363-3 PMC6233260

[23] Baghestan E, Irgens LM, Børdahl PE, Rasmussen S. Trends in risk factors for obstetric anal sphincter injuries in Norway. Obstet Gynecol. 2010;116:25–34. 10.1097/AOG.0b013e3181e2f50b. 20567164 10.1097/AOG.0b013e3181e2f50b

[24] Laine K, Yli BM, Cole V, Schwarz C, Kwee A, Ayres-de-Campos D, et al. European guidelines on perinatal care-peripartum care episiotomy. J Matern Fetal Neonatal Med. 2022;35:8797–802. 10.1080/14767058.2021.2005022. 34895000 10.1080/14767058.2021.2005022

[25] Räisänen S, Selander T, Cartwright R, Gissler M, Kramer MR, Laine K, et al. The association of episiotomy with obstetric anal sphincter injury-a population based matched cohort study. PLoS One. 2014;9:e107053. 10.1371/journal.pone.0107053. 25203655 10.1371/journal.pone.0107053 PMC4159295

[26] Baghestan E, Bordahl PE, Rasmussen SA, Sande AK, Lyslo I, Solvang I. A validation of the diagnosis of obstetric sphincter tears in two Norwegian databases, the medical birth registry and the patient administration system. Acta Obstet Gynecol Scand. 2007;86:205–9. 10.1080/00016340601111364 17364284 10.1080/00016340601111364

[27] Laine K, Pirhonen T, Rolland R, Pirhonen J. Decreasing the incidence of anal sphincter tears during delivery. Obstet Gynecol. 2008;111(5):1053–7. 10.1097/AOG.0b013e31816c4402. 18448735 10.1097/AOG.0b013e31816c4402

[28] Stedenfeldt M, Øian P, Gissler M, Blix E, Pirhonen J. Risk factors for obstetric anal sphincter injury after a successful multicentre interventional programme. BJOG. 2014;121:83–91. 10.1111/1471-0528.12274. 23682573 10.1111/1471-0528.12274

[29] Jurczuk M, Phillips L, Bidwell P, Martinez D, Silverton L, Sevdalis N, et al. A care bundle aiming to reduce the risk of obstetric anal sphincter injury: a survey of women’s experiences. BJOG. 2024. 10.1111/1471-0528.18029. 39663780 10.1111/1471-0528.18029 PMC11879911

[30] Kleprlikova H, Kalis V, Lucovnik M, Rusavy Z, Blaganje M, Thakar R, et al. Manual perineal protection: the know-how and the know-why. Acta Obstet Gynecol Scand. 2020;99:445–50. 10.1111/aogs.13781. 31793662 10.1111/aogs.13781

