# Supplementary figures and images for: Secular Trends in Recurrent Obstetric Anal Sphincter Injuries among 297,190 Women in Norway: A Cohort Study
Source: Int Urogynecol J. 2025 Nov 14;37(5):1307–15. doi: 10.1007/s00192-025-06417-2 (PMC13226332; doi:10.1007/s00192-025-06417-2)

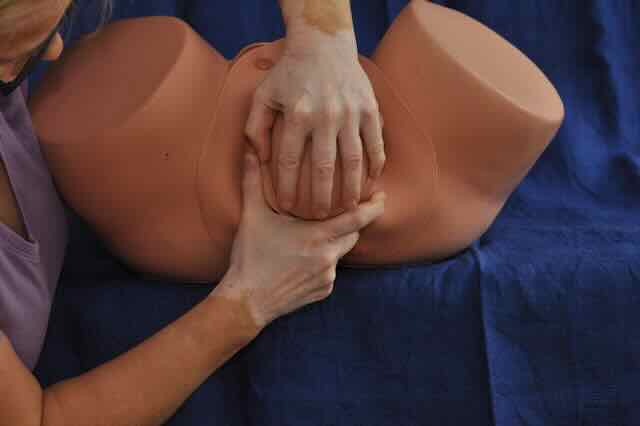

Supplement: Supplementary file 1 — Supplementary Fig. 1 The photo illustrates the methods of manual perineal protection with two hands. (JPEG 13 KB) [file 192_2025_6417_MOESM1_ESM.jpeg]
